# Personalized anticancer therapy selection using molecular landscape topology and thermodynamics

**DOI:** 10.18632/oncotarget.12932

**Published:** 2016-10-26

**Authors:** Edward A. Rietman, Jacob G. Scott, Jack A. Tuszynski, Giannoula Lakka Klement

**Affiliations:** ^1^ BINDS lab, College of Information and Computer Sciences, University of Massachusetts Amherst, Amherst, MA, USA; ^2^ Wolfson Center for Mathematical Biology, Mathematical Institute, University of Oxford, Oxford, UK; ^3^ Department of Integrated Mathematical Oncology, H. Lee Moffitt Cancer Center and Research Institute, Tampa, FL, USA; ^4^ Department of Oncology, Faculty of Medicine and Dentistry, University of Alberta, Edmonton, Alberta, Canada; ^5^ Department of Physics, University of Alberta, Edmonton, Alberta, Canada; ^6^ Molecular Oncology Research Institute, Tufts Medical Center, Boston, MA, USA; ^7^ Pediatric Hematology Oncology, Floating Hospital for Children at Tufts Medical Center, Boston, MA, USA; ^8^ Sackler School of Graduate Biomedical Sciences at Tufts University, Boston, MA, USA

**Keywords:** precision medicine, targeted agents, glioma, topology, thermodynamic measures

## Abstract

Personalized anticancer therapy requires continuous consolidation of emerging bioinformatics data into meaningful and accurate information streams. The use of novel mathematical and physical approaches, namely topology and thermodynamics can enable merging differing data types for improved accuracy in selecting therapeutic targets. We describe a method that uses chemical thermodynamics and two topology measures to link RNA-seq data from individual patients with academically curated protein-protein interaction networks to select clinically relevant targets for treatment of low-grade glioma (LGG). We show that while these three histologically distinct tumor types (astrocytoma, oligoastrocytoma, and oligodendroglioma) may share potential therapeutic targets, the majority of patients would benefit from more individualized therapies. The method involves computing Gibbs free energy of the protein-protein interaction network and applying a topological filtration on the energy landscape to produce a subnetwork known as persistent homology. We then determine the most likely best target for therapeutic intervention using a topological measure of the network known as Betti number. We describe the algorithm and discuss its application to several patients.

## INTRODUCTION

The overriding principle of personalized medicine is the uniqueness of each individual patient, creating an equally unique molecular signature of the patient's cancer. After more than half a century of cancer treatment based on non-specific chemotherapy and radiation, oncologists are beginning to embrace precision oncology as means of incorporating these distinctive, patient-specific cancer molecular signatures. The goal of precision medicine is to select the best, i.e. an individual specific, therapy. It recognizes that based on the genetic and phenotypic background of an individual, a different biological pathway may be engaged in tumor initiation and progression. In most cancers more than one genomic and proteomic alteration is identified exposing our inability to establish the importance of one molecular alteration over another. Much too often the oncologist faced with the dilemma of multiple molecular targets ends up enrolling the patient on one of the available Phase 1 clinical trials, “matching” no more than a single mutation. Not infrequently a patient may be placed on the wrong pharmacological agent if the Phase I trial that targets a less important genomic alteration. Furthermore, because most present clinical trial designs allow for testing of a single agent per trial only, in cases where more than one genomic alteration is present – the other activated pathways will not only remain active, their activity may get further escalated by inhibiting a single target. Most cancer signaling pathways show a large degree of redundancy, which precludes the possibility of finding a “silver bullet”. For successful therapy it is important to recognize and preserve the eco-evolutionary forces within the tumor microenvironment [1]. It is therefore critical to develop methods that can analyze the varied interaction of the majority if not all of the detected genomic alterations. The use of such a pathway-analysis based model, which studies the individual tumor signatures within the context of well-established protein-protein interaction (PPI) networks may prevent us from inadvertently placing our patients on therapies targeting the less-than-optimally effective of the molecular alterations.

These protein-protein interaction networks reflect cell dynamics, and mirror the well-coordinated and controlled interactions within a cell. A set of all known interactions gives rise to a network, which can be characterized and analyzed using rigorous mathematical and physical methodology. A state-of-the-art example of a PPI network database is Biogrid (http://thebiogrid.org), first described by Breitkreutz et al. in 2002 [2], and subsequently updated [3]. These academically curated PPI networks, despite being continuously updated and representing the state-of-the-art today, have not yet been completely mapped out from the presently available open-reading frames of genes and proteins. As such, any calculations of thermodynamic properties done on a today's network can represent only an estimate that reflects the present state of knowledge about protein-protein interactions.

That said, the effort currently being exerted on developing mathematical, physical and biological methods for selecting the best therapeutic target is not in vain and allows for rational implementation of new cancer cell biology discoveries which may result in the development of future therapeutics. We describe a novel methodology for using the extensive amounts of online bioinformatics data to analyze high-throughput genomic information from a patient's tumor (in this case RNA sequence data) to improve the quality of present-day clinical decisions. The application of mathematics in molecular systems biology, and the descriptions and conjectures about the application of group theory and abstract algebra in molecular networks, have been well-reviewed previously [4, 5]. For example, some of the present authors recently found that the entropy of protein-protein interaction (PPI) networks is well correlated with 5-year patient survival [6], making findings at the level of subcellular networks of relevance to clinical applications. Similarly, an abstract mathematical concept known as the cardinality of the automorphism group, when applied to cancer protein-protein interaction networks also correlated with patient survival [7], enabling predictions about the potential gains in patient survival as a result of optimized target determination. The accuracy of these predictions can be further enhanced through the use of additional topological measures such as Betti number or homology of cancer PPI networks [8] as is elaborated on below.

We surmise that the eventual development of reliable clinical tools enabling clinical oncologists to correctly analyze and apply an individual's genomic information is likely to require a much wider array of mathematical and physical tools, but we contend that the best present computational approach should involve a combination of Gibbs free energy [8, 9] and homology [10], an approach we refer to as Gibbs homology. It is important to clarify that while the term “homology” may be used by molecular biologists and biochemists to describe *similarity* in protein sequence or structure; mathematicians use this term to describe similarity in topological surfaces. As such, the term persistent-homology describes a topological surface feature that is observed despite the presence of noise or surface roughness. In this manuscript, we use the term persistent homology as a topological measure on Gibbs free energy surfaces for protein-protein interaction networks. We combine the concept of Gibbs energy on PPI networks to compute the persistent homology, and then use Betti number to compute a therapeutic target or targets according to a set of rules described below.

In the subsequent paragraphs, we introduce the theoretical background for this method, using the extensive microarray and RNAseq transcription information available in The Cancer Genome Atlas (TCGA, http://cancergnome.nih.gov). We will introduce a novel method for superimposing transcription information from individual patients (or the extensive RNAseq data available through The Cancer Genome Atlas - TCGA - http://cancergnome.nih.gov) onto a human protein-protein interaction network with the goal of selecting targets for therapeutic intervention.

## RESULTS AND DISCUSSION

Low-grade glioma (LGG) is an ill-defined subgroup of glial tumors, associated on the basis of their clinical features (slow growth) and some histological features (GFAP and low proliferative fraction). There are three main histological subtypes: low grade astrocytoma, oligoastrocytoma, and oligodendroglioma. While these may represent very distinct molecular entities, their biological behavior is sufficiently similar for this grouping. Most importantly, because these slow growing tumors have a very low proliferative fraction and do not respond to chemotherapy and radiation, there are no existing effective therapeutic interventions. The identification of molecularly guided targeted therapies, which do not depend on the proliferative fraction in the tumor, would provide viable therapeutic strategies for poor prognosis cancers such as DIPG or low-grade thalamic gliomas.

**Figure 1:**
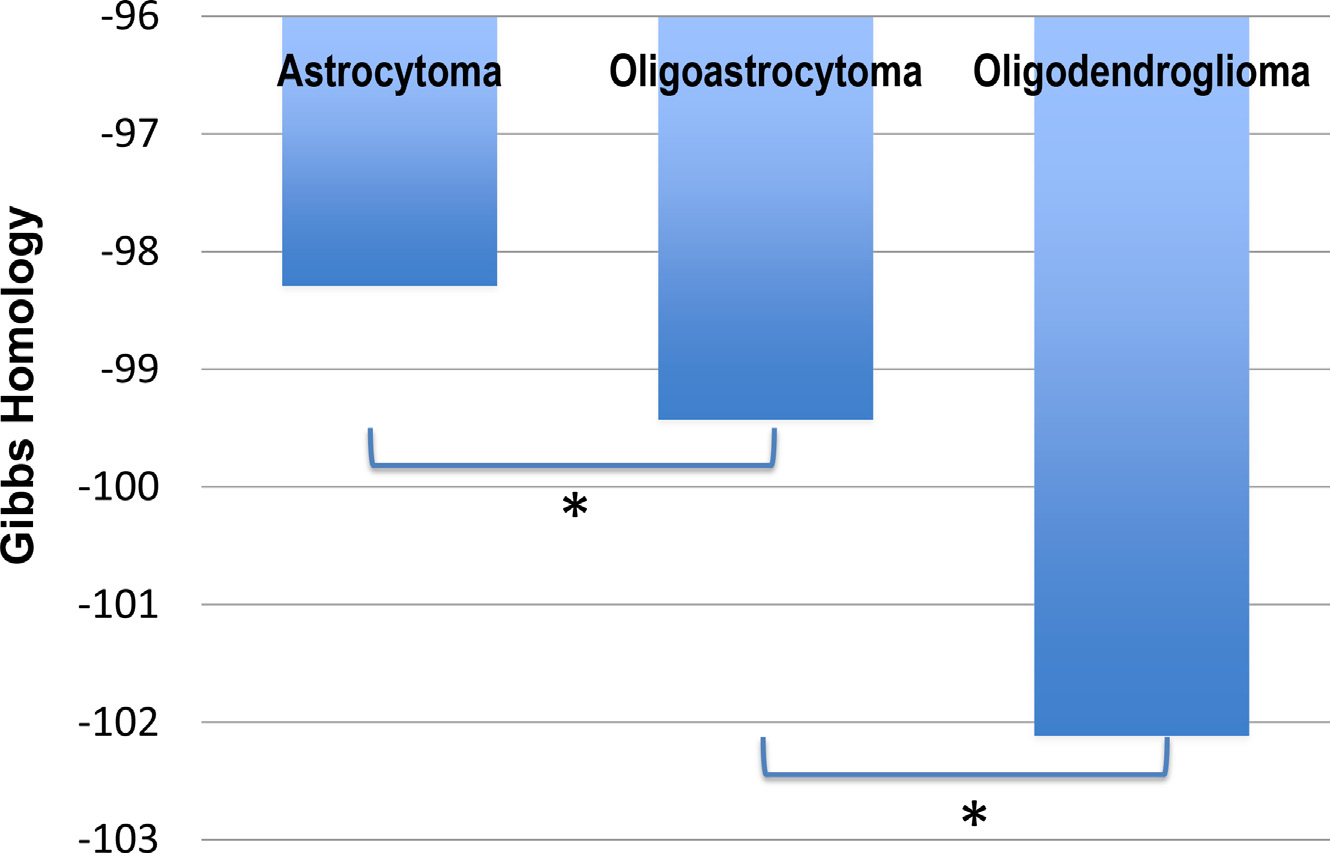
Gibbs free energy for The Cancer Genome Atlas (TCGA) patient data for Low Grade Glioma (LGG) The TCGA contained data of 194 patients with astrocytoma, 129 patients with oligoastrocytoma, and 191 patients with oligodendroglioma (total low grade gliomas 514). We found significant difference in the average total free energy (Gibbs homology) between astrocytoma and oligoastrocytoma (*p* = 0.0746), and between oligoastrocytoma and oligodendroglioma (*p* < 0.0001). As such the clinical observation of poorer survival in oligodendroglioma is reflected in the increased entropy (increasingly negative free energy) of these cancers.

Using the TCGA data we computed the Gibbs free energy for the homology subnetwork of each patient with LGG, and found statistically significant differences in energy between the three different cell types (Figure 1). The Gibbs free energy of oligoastrocytoma is significantly different from oligodendroglioma (*p* < 0.0001), and there is a trend to statistically significant difference between astrocytoma and oligoastrocytoma (*p* = 0.0746).

**Figure 2:**
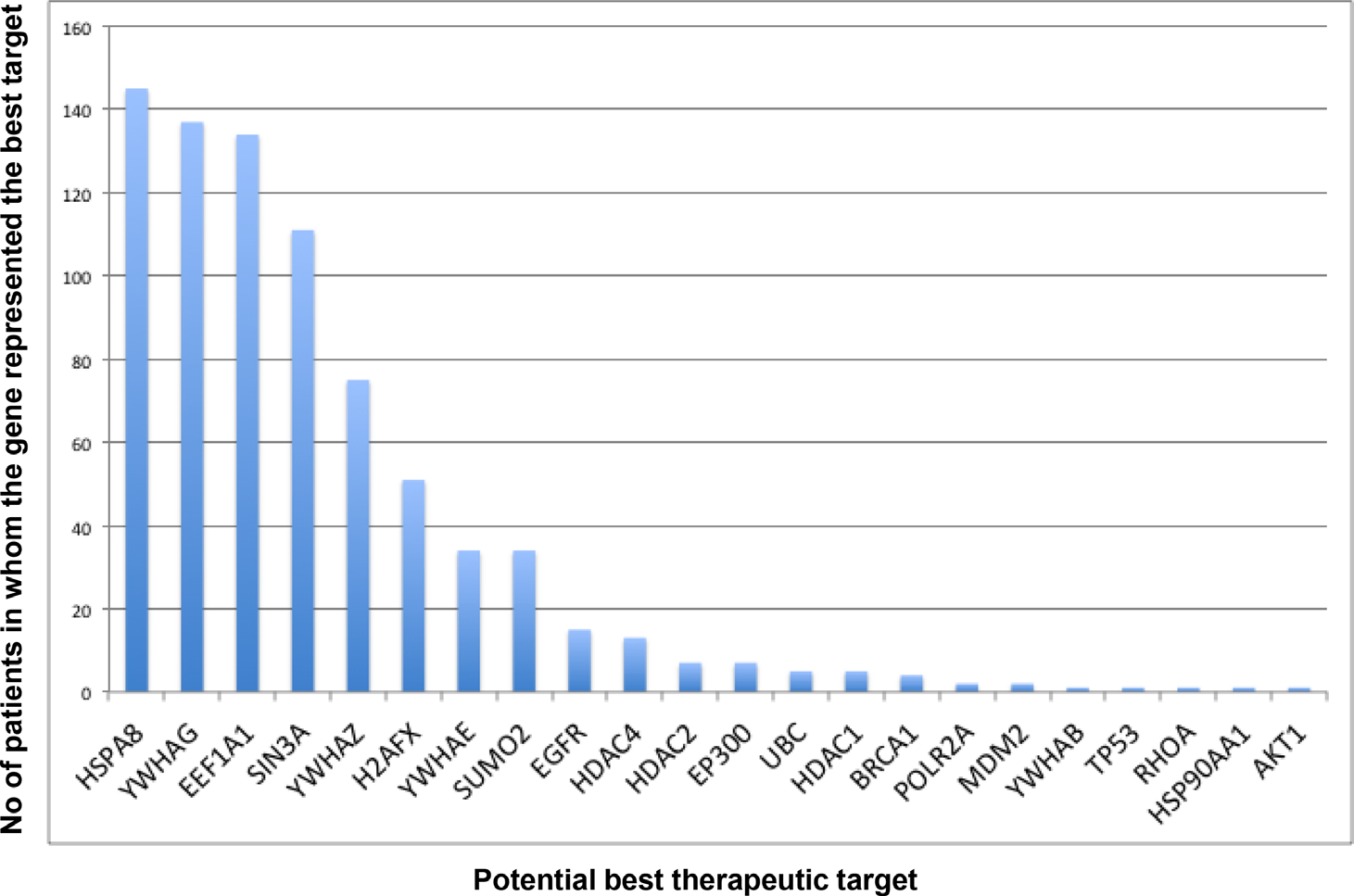
Pareto chart of the computed potential therapeutic targets Potential therapeutic targets were derived by first computing the Gibbs homology at energy threshold of 32 followed by sequential deletion (with replacement) of individual proteins. The proteins, which when deleted produced the biggest reduction in Betti number, are listed as the potentially therapeutic targets. The chart suggests that HSPA8 (a heat shock protein) is the most common “target” being present in 145 patients out of the total 514, but it does not represent the best target in 72% of the patients (369/514). This is a very important observation suggesting that a successful treatment of glioma requires a more personalized approach in majority of patients.

A Pareto chart ranking the “best therapeutic targets” for these 514 patients with LGG available from TCGA (Figure 2) shows that the most frequent best target in 145/ 514 patients is novel. While HSPA8 (Heat Shock protein 8) has been associated with spinocerebellar ataxia type 17 in the past, it has not been considered a target for glial cancers. Similarly, YWHAG (Tyrosine 3-Monooxygenase/Tryptophan 5-Monooxygenase Activation Protein, Gamma) appears to be the best target for 137/ 514 patients with LGG, and even though it is an active participant in the signaling through the RAF1, AKT1 and Jun oncogenic pathways, it has not been considered to be a therapeutic target in gliomas. Consequently, these two proteins represent novel targets for a substantial fraction of the patient population analyzed in our study. The other bars in the chart can be interpreted similarly. Recall that we identified the potential therapeutic targets by deleting each individual node (protein) from the Gibbs homology subnetwork at energy threshold of 32, and finding the one protein that produced the most significant change in complexity. Because there may be more than one equivalent target (those with equivalent drop in complexity associated with different proteins), the sum of all potential therapeutic targets is higher than the total number of patients (in this case 786 targets for 514 patients, Supplementary Table S1).

Interestingly, the analysis of the homology networks for patients in whom elimination of HSPA8 leads to most significant changes in complexity revealed similarities in the homology subnetworks despite the differences in tissue type. This is likely due to the observation that the protein neighbors of HSPA8 within a PPI network are the same in the different tissue types (astrocytoma, oligodenroglioma and oligoastrocytoma) and the expression of HSAP8 is determined by the degree entropy rather than by the tissue type. For example, the astrocytoma patient (Patient ID 2 in Table 1) had the following protein interaction neighbors to the HSPA8: SUMO2, TP53, UBC, RHOA, EGFR, APC, HSP90AA1, and YWHAG, consistent with the published and well documented signaling pathway interactions of HSPA8. The patient with oligoastrocytoma (Patient ID 294 in Table 1) had the identical set of protein interaction neighbors. In contrast, the patient with oligodendroglioma (Patient ID 8 in Table 1) had, in addition to the same identical set of neighbors, PARP1 as an additional neighbor suggesting recruitment of an additional signaling pathway. As the overexpression of PARP1 becomes sufficient to pass the energy threshold of 32, the change in Gibbs free energy is reflected in the different targets in patient ID 2 and patient ID 8. We provide in Supplementary Table S1 patient IDs, Gibbs energy for the homology network for the whole network, entropy for the subnetwork and for the whole network, Betti numbers for the numbers for the subnetwork, number of edges, etc. but we do not identify the respective neighbors.

**Table 1:** Four examples of patients with different types of low grade glioma

Histologic_diagnosis	Days-to-death	TCGA ID (Patient ID)	Entorpy	Nominal betti	Gibbs of homology net	Gibbs of whole network	Number of edges in homology net	Change in Gibbs after target removal	Targets and reduced Betti
Astrocytoma Gr3	1335	TCGA-CS-4942 (Patient 2)	2.461440845	20	−94.37907586	−6206.432916	130	−26.50085696	(u′HSPA8′,15)
Oligodendroglioma Gr2	639	TCGA-CS-5395 (Patient 8)	2.64534633	28	−100.465829	−6571.397908	140	−28.44663419	(u′HSPA8′,22) (u′H2AFX′,22)
Oligodendroglioma Gr2	N/A	TCGA-FG-8182 (Patient 203)	2.826529111	23	−99.2066031	−6551.667165	144	−27.99459365	(u′HDAC4′,18) (u′HSPA8′,18)
Oligoastrocytoma Gr2	1933	TCGA-HT-8013 (Patient 294)	2.663861005	26	−94.90096615	−6257.933862	150	−26.62233376	(u′EEF1A1′,21) (u′HSPA8′,21)

**Figure 3:**
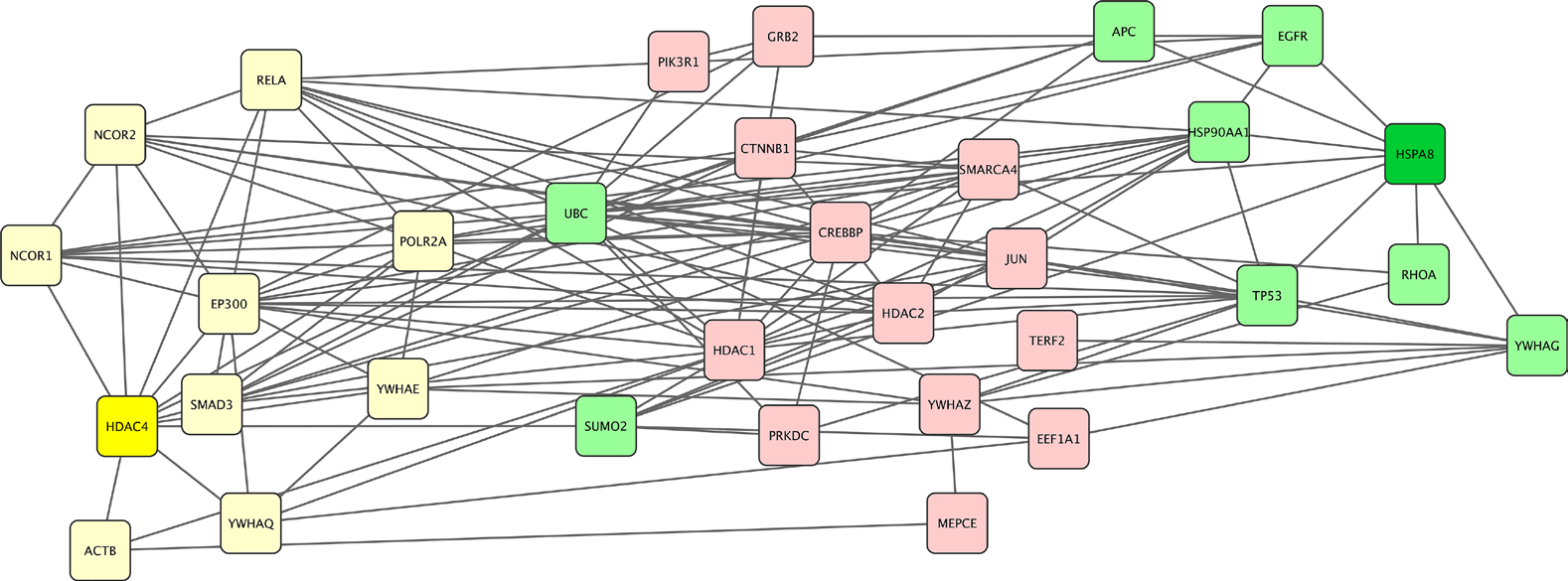
Gibbs homology subnetwork example for a single patient For a patient with oligodendroglioma (patient ID #203 in Supplemental Table S1]. The algorithm found two equivalent targets of the homology subnetwork at energy threshold 32. Both of these targets, HDAC4 and HSPA8 are shown in dark yellow and green respectively with the lighter colors indicating their respective neighbors. The remaining proteins represent non-neighbors and are labeled pink.

As we have shown, many patients with distinct glioma subtypes had HSPA8 computed as the best target, suggesting they may be treated with the same agent that inhibits of silences this particular protein. However, the actual selection of this therapeutic target involves additional bioinformatics analysis. Using HSPA8 as a therapeutic target in patient ID 203 with oligodendroglioma (Table 1) would not be sufficient because there were two equivalent therapeutic targets - HSPA8 and HDAC4. The proteins interacting with HSPA8 (SUMO2, TP53, UBC, RHOA, EGFR, HSP90AA1, YWHAG) and the set of proteins interacting with HDAC4 (UBC, YWHAE, RELA, NCOR1, SUMO2, YWHAQ, NCOR2, SMAD3, EP300, POLR2A, and ACTB) overlap in only two proteins (SUMO2 and UBC) and their pathways are not identical. While the neighborhoods of HSPA8 and HDAC4 have similar Gibbs free energy, because they were defined at the same energy threshold, they will require different therapeutic modulation. Figure 3 shows the homology subnetwork for patient #203 with oligodendroglioma. Note that some proteins are common to both sets - UBC and SUMO2 suggesting the involvement of proteasome/ubiquitin pathways. Again, while this pathway has been the favored target in multiple myeloma, it has not been explored therapeutically in glioma. HSPA8 and HDAC4 proteins are colored dark green or yellow, respectively, the neighbors of HSPA8 are green and the neighbors of HDAC4 are yellow. All other proteins are light pink.

The protein neighbors of HSPA8 and HDAC4 have similar, albeit not identical, Gibbs free energy and form part of the persistent homology, as the inhibition of HSPA8 or HDAC4 can drop the network complexity (Betti number) by a comparable amount. Because reducing complexity or Gibbs free energy can be correlated with increased probability of 5-year survival [6, 8], we expect that Gibbs free energy reduction on the homology subnetwork will also correlate with improved survival, but the evidence remains to be clinically evaluated. For now, these complexity measures can be used to gain insights into these pathways and assist clinicians in making informed decisions regarding therapeutic options, especially if they can reply on either approved drugs for a given type of cancer or the use of off-label medications which would eliminate the costly and time-consuming process of novel drug development.

In order to determine what role HDAC4 and HSPA8 along with their similar energetic neighbors play in cancer, we submitted the list of proteins to DAVID (https://david.ncifcrf.gov/) and KEGG (http://www.genome.jp/kegg/). This allowed us to obtain the disease pathways known to be associated with these proteins. We found for example, EGFR is involved in colorectal cancer, pancreatic cancer, endometrial cancer, prostate cancer, melanoma, bladder cancer and non-small cell lung cancer, and it has been previously considered a good therapeutic target in glioma [11, 12] chiefly because it is associated with infiltrative gliomas [13]. The importance of TP53 in glioma is well established and not dissimilar from its importance in colorectal cancer, pancreatic cancer, endometrial cancer, prostate cancer, thyroid cancer and basal cell carcinoma. However, p53 is mutated in almost 50% of all cancers and there is a dire need to develop activators of mutated forms of this protein. In contrast, RELA, which has come up in a number of glioma molecular studies [14, 15] has as of yet not been thought of as a therapeutic targeted. Yet, its role in progression of prostate cancer, pancreatic cancer, chronic myeloid leukemia, acute myeloid leukemia and small cell lung carcinoma suggests a strong therapeutic potential. One can make the same argument for all the neighbors of HSPA8 and HDAC4, some of which are of known significance and others new to the role. The role of RHOA in cancer, even though listed in “pathways to cancer” in KEGG, is unknown. SMAD3 is involved in colorectal cancer, pancreatic cancer and chronic myeloid leukemia, APC is involved in colorectal cancer, endometrial cancer and basal cell carcinoma, and HSP90AA1 in prostate cancer, but none of these would be considered as targets by a neuro-oncologist. While HDAC4 expression has been used to identify patients likely to respond to temozolomide or radiotherapy [16], and a high HDAC4 expression is a signature of low-grade glioma [17, 18], it has not been viewed as having a direct therapeutic value to date. Yet, chromosomal instability quantified by HDAC expression can be closely correlated with clinical outcomes in many cancers [18], and more specifically in glioma [19]. In summary, this relatively simple analysis of Gibbs Homology, Betti-defined proteins and their energetic neighbors may help identify potentially effective therapeutic targets, some of which may have been previously identified in other cancers, and some of which will be entirely new.

The most important point made by this thermodynamic analysis of TCGA data on low grade glioma is that very similar pathways can be employed by tumors of different histological features. While all cells of the human body may have the same set of genes, each cell uses a very different set of genes to facilitate its physiological maintenance and growth. The gene sets involved in the growth and progression of a cell that has undergone malignant transformation may be very different from the normally engaged genes as developmentally dormant pathways may be used. Some of these developmentally silent genes may be more frequent than others. Out of the 514 patient cancers investigated in this paper, 145 used HSPA8 as its main mechanism of action and 369 employed an alternative pathway. Yet, while the frequency of HSPA8 gene alteration may be sufficient for a successful clinical trial, it may not be of benefit for most patients. This underscores the importance of having individualized data when making therapeutic decisions. For a patient, the statistics are simple, their tumors either do employ a pathway, or they do not.

## CONCLUSIONS

We have explored the potential of overlaying real transcription information from a patient's tumor on academically curated protein-protein interaction networks to define energetically important nodes. The use of these thermodynamic and topological measures enabled realistic selection of potential therapeutic targets, because the combination of the two topological concepts (i.e. landscape filtration, called here an energy threshold) and Betti numbers (which quantify the presence of rings in a network) can identify the pathways most important for the growth and dissemination of the patient's tumor. It is likely that in a not-too-distant future, similar mathematical and physical tools may be used prospectively for designing biopsy-guided therapy. However, as we show, a retrospective analysis of genomic profiles of patients enrolled in clinical trials may reveal which patients were exposed to therapeutically appropriate targets, and provide a unique window of understanding for our failures to successfully implement targeted therapies. It is likely that with careful retrospective analysis, many “failed” agents may be returned to our pharmacopeia, because even though some patients may have a therapeutic target similar to other patients, most patients have a unique molecular signature requiring a *combination* of optimally selected therapeutic agents. The recent emergence of precision medicine [20, 21] represents recognition of this new therapeutic trend.

## THEORETICAL BACKGROUND

**Figure 4:**
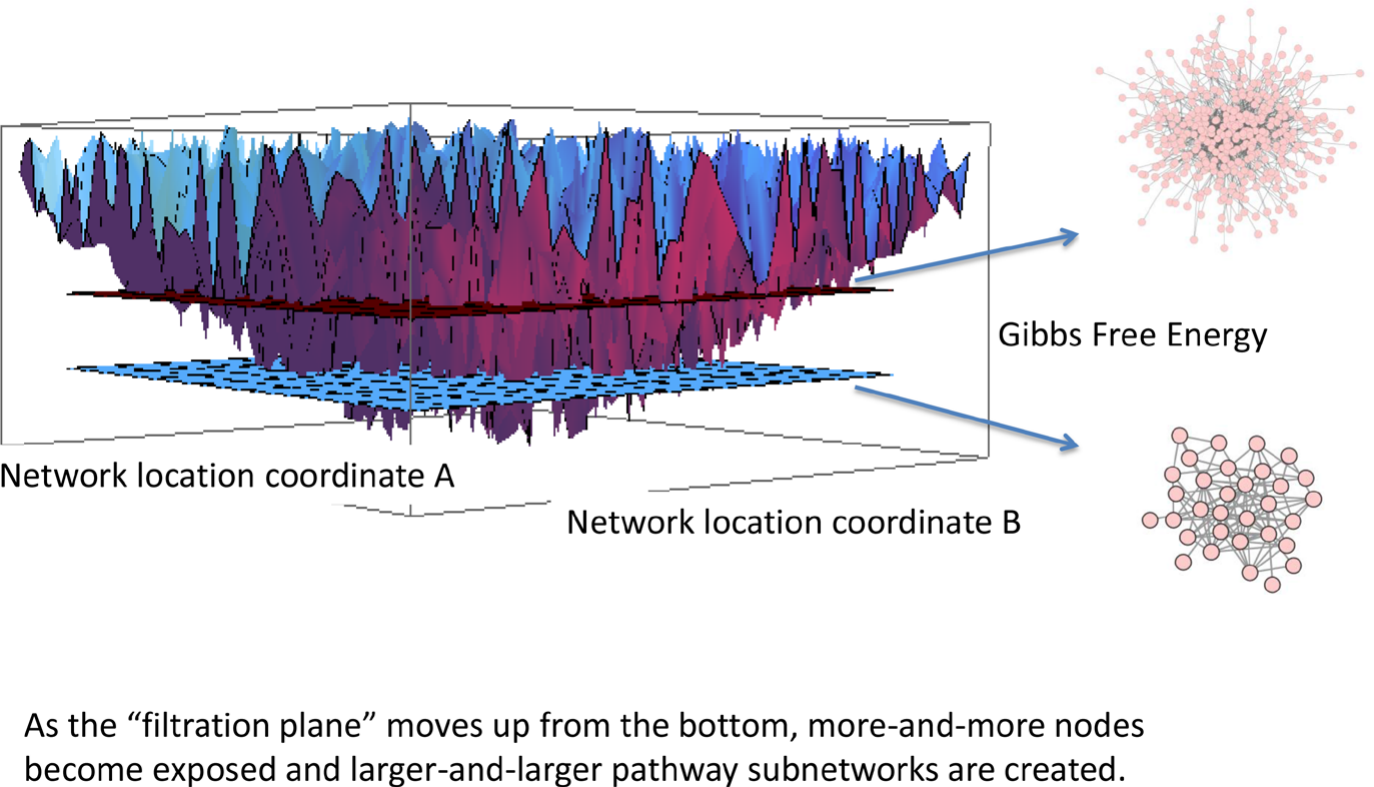
Filtration of energy landscape of a protein-protein interaction network (PPI) Each of the energy wells represents an individual protein in the human- scale PPI. The deeper the well, the lower the free energy and the higher the entropy. Gibbs free energy is a negative number representing the concentration of the protein *and* the degree entropy. By sliding a filtration plane up from the deepest well, the plane intersects gradually with a greater number of energy wells, and because the energy landscape is an abstraction of the PPI network, the Gibbs free energy “captures” a different number of nodes in the network at each level. Thus, at deeper energy thresholds a much lower number of nodes is captured, and at higher energy thresholds more and more nodes are captured. The subnetworks captured at the different energy thresholds are persistent homology, and we show two such networks for illustrative purposes.

The energy between interacting molecules (interaction energy) is referred to in statistical thermodynamics as a chemical potential. Whenever the concentration of one molecular species changes, the reactions in which this molecular species participates is therefore affected as well. Thus, a change in one protein concentration will percolate through the network affecting multiple other molecular species, and changing the network's chemical potential. The concept is applicable to any large-scale molecular network, including established biological protein-protein interaction networks such as Biogrid^®^. The energetic state of a cell can therefore be described by the sum of the chemical potential (Gibbs free energy) of all interacting pairs of proteins within the network; and the perturbations and variations in chemical energy can be graphically represented by a rugged energy landscape such as the one illustrated in Figure 4. Hence the chemical energy of a PPI network can be represented as an energy landscape [22, 23].

We have previously described how to use RNA transcription data and protein- protein interaction data to compute the Gibbs free energy for various types of cancers represented by their PPI networks [8, 9]. It should be stressed that the collective RNA transcriptome can still be informative about the relevant biological aberrations with the caveat that the total RNA extracted from a tumor tissue represents an average of a very heterogenous collection of cells, and embodies not only the actual cancer clone(s), but also the activated host stroma. The transcriptional information provides a good estimate of protein concentration, as the increased/ decreased gene transcription (whether due to mutational activation or due to stromal induction) invariably leads to an increase in protein production. This has been substantiated in past investigations where it was found that correlations between mRNA and protein concentrations in a large number of experiments across five different species spans the range of 40–80% [24, 25]. Similarly, a separate study found the correlation between mass spectrometry proteomic information and transcriptomic information to be 83% for multiple tissue types [26, 27]. While there is an emerging need for caution as the recent emergence of RNA-seq replaces gene expression microarrays [28], the multiple sources of transcription data contained in TCGA can be corrected for the different sets of biases. The differential expression data from microarrays may be biased by the need to decide *a priori* what content to place on the arrays, the RNA-seq, which does not use probes or primers, needs to convert mRNA (and other RNAs) to cDNA, which is then used as the input to a next-generation sequencing library. The RNA-seq has the ability to uncover new and rare driving mutations, but it is only as good as the ability to use the most appropriate library. Until it is established that RNA-seq derived transcription information is comparable to microarray methods, and until there is a sufficient number of studies comparing gene expression levels by microarrays vs. those obtained using RNA-seq, the two may be best analyzed separately. Yet, a large-scale comparison of gene expression levels by microarrays and RNA-seq using TCGA data for fourteen different cancer types found a Spearman correlation of about 0.8 [29], and the comparison of Affymetrix microarray data to the Illumina RNA-Sequencing technology using breast cancer data revealed a correlation of 0.7 [30], which gives a certain amount of confidence in the use of RNA-seq data for PPI network analyses.

Given the close correspondence of microarray and RNA-seq data, we can use either of these data types as surrogates for protein concentration estimates. Koussounadis et al. [31] show that differentially expressed mRNAs correlate significantly better with their protein product than non-differentially expressed mRNAs, mainly because of abundance [32]. These studies increase confidence for the use of differential mRNA expression for biological discovery and for inferences from mRNA expression. While the correlation of RNA levels and protein levels is over 80% [31, 32], it should be recognized here that the degree of activity of a protein due to post-translational activation cannot be estimated at the present time. We recommend that whenever possible an actual estimate of protein activity (rather than expression) be used for Gibbs Homology calculation.

We combine this surrogate “protein concentration” with the PPI and create an energetic landscape represented by chemical potential of protein-to-protein interactions (Figure 4). Complex dynamical systems such as biological cells exhibit ordered (stable) dynamics under normal physiological conditions, but these complex landscapes may be subject to dynamic changes with any fluctuations in cell phenotype [33]. For example, the trajectory of a transcriptomic landscape following exposure of human promyelocytic leukemia cells (HL60) to either dimetholsulfoxide or all-trans-retinoic acid suggests that cell fates represent high-dimensional attractor states. While we will not explore the trajectory of homogeneous cell populations, we will draw heavily on this energy landscape analogy.

We compute Gibbs free energy for a given cellular population as described previously [9, 10]. Then, making use of hierarchical decomposition of critical nodes of a network [34], we compute persistent homology of the network. The function computes Gibbs free energy, a real negative number for which the smaller the value (larger negative), the deeper the minima in the energy landscape. More specifically, the Gibbs free energy for an individual node, *i*, or individual protein, is given by:

$$G_{i}=c_{i}\ln\frac{c_{i}}{\sum {}_{j}c_{j}}$$ Eq (1)

where the *c* i is the concentration of the protein, or the normalized (rescaled between 0 and 1) expression of protein *i*. The sum in the denominator of Eq. (1) is taken over all nodes *j* in the neighborhood, including *i*. The natural logarithm of this dimensionless number in conventional chemical thermodynamics represents the Gibbs free energy for an individual protein in the network [9]. The overall Gibbs free energy for the entire network is found by summing over the nodes: $G=\sum {}_{i}G_{i}$

Each node in the network has an associated real negative number representing its respective Gibbs free energy creating a schematic of a complex network mapped onto a rugged landscape (Figure 4). Persistent homology is the *persistent* landscape structure that remains even in presence of noise, but the concept remains valid even in absence of noise. Consider a horizontal plane (a filtration plane) moving upwards from the bottom as depicted in Figure 4. At a low level there are only a very few deep minima, but as the *filtration plane* moves up, it cuts through more and more energy wells, *capturing* increasingly more minima. Since these energy wells represent Gibbs free energy of the network, the small sets of energy wells captured by the filtration plane at each energy threshold represent specific subnetworks. The subnetworks, which represent the deepest (highest absolute value) of Gibbs free energy, are called a *persistent ho*mology, and we will hereinafter refer to these energetic subnetworks as *Gibbs homology* networks.

We can now use the analogy of flooding a landscape as a way to intuit the dynamics of energy landscapes, a concept well explored in the past [35]. The deepest minima will be filled first, followed by the next deepest, etc. These deep minima represent the energy minima of a persistent homology, where neighboring wells are closely related in depth, or in this case, closely related in energy.

## DATA SOURCES AND METHODS

We collected RNA-seq data for low grade glioma (LGG) from The Cancer Genome Atlas (TCGA) hosted by the National Institutes of Health (http://cancergnome.nih.gov), and well-described in the past [17]. We focused on the LGG dataset [36] of the human protein-protein interaction network (*Homo sapiens*, 3.3.99, March, 2013) from BioGrid (http://thebiogrid.org) containing 9561 nodes and 43,086 edges [3, 37], and used the adjacency list (i.e. the matrix of protein-protein interactions) of this network for the calculations. For computing the Gibbs free energy we used Python 2.6.4 with networkX functions.

The workflow for the calculations of the Gibbs-homology and determination of the potential protein target(s) for therapeutic intervention is shown in the flowchart of Figure 2. Starting with the relevant RNA-seq (or microarrays) transcription data and the human scale PPI we computed the Gibbs free energy for every node and for the total network according to Equation (1). Note that there is a necessary re- scaling of the RNA expression data into the 0 to 1 range (not shown in the flow chart), which is done as follows: the RNA-seq data are typically normalized counts that can be converted to log2 using the formula: log2(normalized count + 1). This conforms the RNA-seq data to a comparable scale of the microarray transcription data, which are typically log2 normalized. The rescaling of either type data is done by first finding the minimum (e_min_) and maximum (e_max_) of the individual patient dataset, and the log transformed data for each gene, ei in that RNA-seq or transcriptome vector is then processed as:

$$c_{i}=(e_{i}-e_{\min})/(e_{\max}-e_{\min})$$

where *ci* represents the rescaled value and is used in Eq (1).

This rescaling is justified from both a mathematical perspective and a chemical physics perspective. Negative values in the argument of the natural logarithm of Eq. (1) are undefined, and the argument from a chemical physics perspective is based on concentrations. If a gene is very strongly down-regulated (i.e. there is a very low RNA expression), it is unlikely to produce much protein and as such we can assign the protein concentration to zero for the most down-regulated gene in that patient's dataset. In contrast, when a gene is highly up-regulated, or has a high RNA count, it is likely to be translated into a great deal of protein. The rescaling assigns a value of 1 to the highest up-regulated gene (i.e. highest concentration of protein corresponding to this gene).

**Figure 5:**
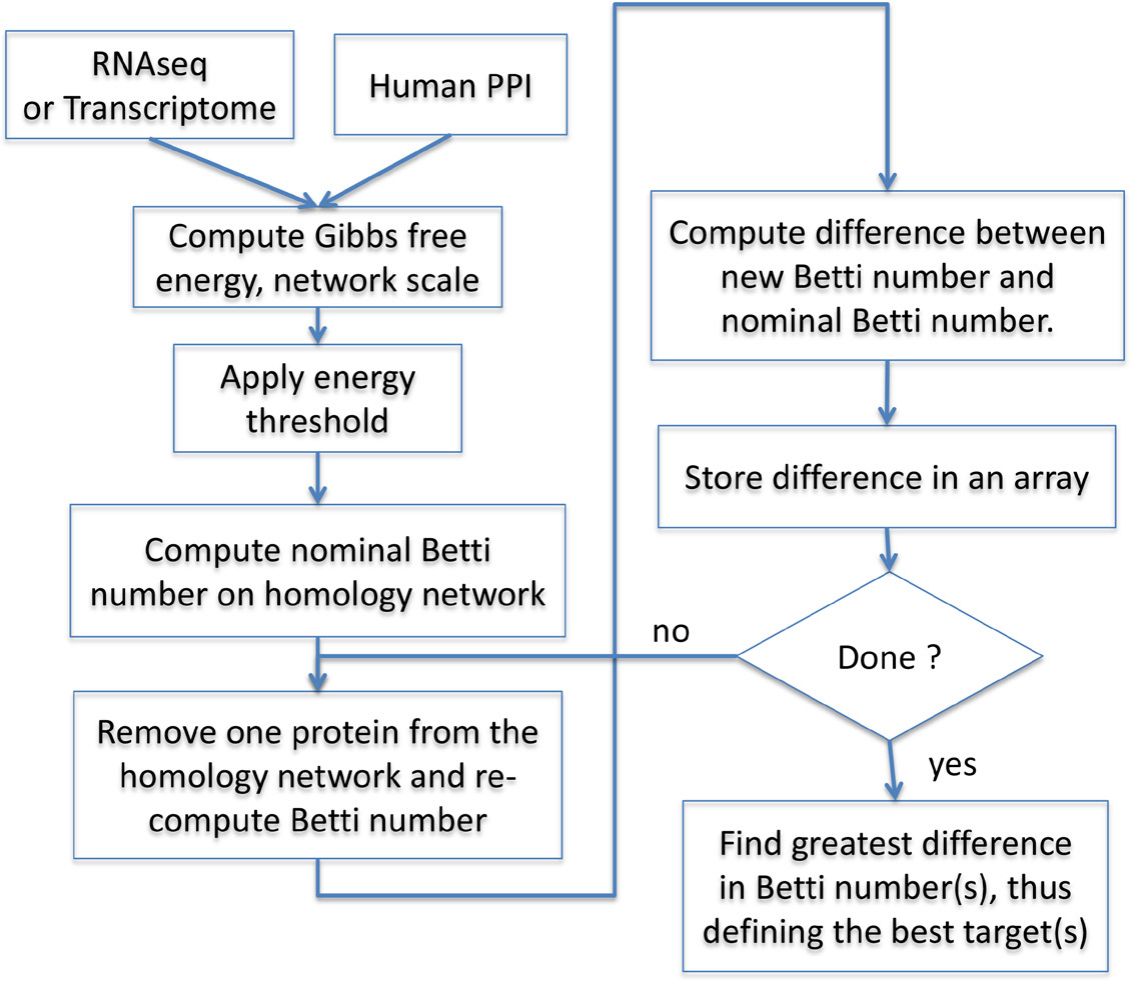
Basic operations flow RNAseq or microarray-based transcription data are used as surrogates for protein concentration. The data are overlaid on the human protein-protein interaction network (BioGrid^®^) and Gibbs free energy of each node is computed according to Equation (1). An energy threshold is then applied to produce the Gibbs homology subnetwork, and Betti numbers are computed for all nodes in this subnetwork. Each node in the subnetwork is then sequentially removed with replacement, and the Betti numbers re-computed following each removal. The outcome is an array containing all of the relevant proteins with their respective Betti numbers. The array can be searched for proteins, which, when removed lead to the greatest drop in Betti number. If the same drop in Betti is achieved by removal of more than one protein, two or more protein targets may be considered equivalent.

The rescaled dataset is then analyzed at a set energy filtration threshold (Figure 5). The selection of the particular energy filtration threshold is done as a simple optimization between having too many potential therapeutic targets that would make a clinical application impossible, and between having inadequate coverage of relevant therapeutic targets. The filtration threshold selection is a one-time task and is based on the Betti number [8]. The Betti number represents the number of rings in a network consisting of four or more proteins with a well-documented interaction within the network. If the removal of a node (protein) from the network results in a large drop in the Betti number (hence a substantial reduction in the number of large enough rings), it reflects a large reduction in network complexity caused by a node removal. Thus, the change between nominal Betti calculated for the entire homology subnetwork, and the Betti calculated after a protein is removed, is a good estimate of the importance the protein has in maintaining the network complexity. The node(s) with the highest drop in complexity should be considered a good therapeutic target(s), because lowering network complexity is associated with improved survival of patients with cancer [8]. This node should then correspond to the most optimal protein target for inhibition by a pharmacological agent. The best therapeutic target is therefore identified when both of the above conditions are met, i.e. when enough complexity (determined by the energy filtration threshold) is present, and a large enough drop in complexity (determined by Betti number) has been achieved by inhibiting the target.

When the energy threshold is low (e.g. 8, 16), the complexity of the subnetwork is also low, and removing any individual protein will drop the Betti number by the same amount resulting in as many as eight or more equivalent targets. In contrast, at high-thresholds (e.g. 64, 128) typically only one node leads to a large drop in the Betti number. The energy filtration threshold was optimized by identifying the best targets through a systematic (sequential) application of energy thresholds between 8 and 128 (i.e. 8, 16, 32, 48, 64, 128). We found the most clinically relevant information is to be derived at threshold of 32, where an average of about 1.514 equivalent targets (SD+/− 0.915) is found. At this level, for the majority of patients one or two potential targets are found, but there may be up to three equivalent targets. This provides a good margin for selection of treatment options aimed at inhibiting these proteins as targets for pharmacological agents.

At each threshold, and for each patient in the population of patients, we computed Gibbs homology, total Gibbs energy (nominal energy) for the homology subnetwork, and the nominal Betti number for this Gibbs homology. The information for each patient in the set therefore included Gibbs homology subnetwork, Gibbs energy, and Betti number. We then sequentially removed (with replacement) each protein from the Gibbs homology subnetwork, recomputed the total Gibbs energy of the subnetwork, and determined the respective change in Betti number. This provided us with an array of protein targets and their respective changed Betti numbers, as well as the respective change in Gibbs energy for each patient in the set. By searching this array for the smallest Betti number (the most reduced Betti), which corresponds to the biggest reduction in complexity of the homology subnetwork we could make specific personalized predictions for the best potential therapeutic targets. We re-iterated this process for each patient in the set, and kept track of the lowest Betti number(s) and respective target(s) (See Table 1).

## SUPPLEMENTARY MATERIALS FIGURES AND TABLES




