# Supplementary material for: Personalized anticancer therapy selection using molecular landscape topology and thermodynamics
Source: Oncotarget. 2016 Oct 26;8(12):18735–45. doi: 10.18632/oncotarget.12932 (PMC5386643; doi:10.18632/oncotarget.12932)
Supplement: Supplementary file 1 [file oncotarget-08-18735-s001.pdf]

Personalized anticancer therapy selection using molecular landscape topology and thermodynamics

Supplementary Materials

Supplementary Table S1: Complete Summary of the 514 TCGA patients analyzed.
